# Indole-3-propionic acid alleviates chondrocytes inflammation and osteoarthritis via the AhR/NF-κB axis

**DOI:** 10.1186/s10020-023-00614-9

**Published:** 2023-01-31

**Authors:** Huangming Zhuang, Xunshan Ren, Fuze Jiang, Panghu Zhou

**Affiliations:** Department of Orthopedics, Renmin Hospital of Wuhan University, Wuhan, 430060 China

**Keywords:** Indole-3-propionic acid, Osteoarthritis, Aromatic hydrocarbon receptor, Chondrocytes, Inflammation

## Abstract

**Background:**

Osteoarthritis (OA) is a common chronic disease characterized by chronic inflammation and extracellular matrix degradation. Indole-3-propionic acid (IPA) is a tryptophan metabolite secreted by intestinal flora, which can exert anti-inflammatory effects in a variety of diseases. In this study, we further investigated the potential therapeutic role of IPA in OA and the underlying mechanism.

**Methods:**

IL-1β was utilized to induce chondrocyte inflammation. Then, the cytotoxicity of IPA on rat chondrocytes was assessed. Meanwhile, RT-qPCR, Griess reaction, ELISA, Western blot and immunofluorescence were performed to evaluate the expression of inflammatory factors and stromal proteins, and the NF-κB pathway in chondrocytes treated with IL-1β alone, with IPA or with aryl hydrocarbon receptor (AhR) knockdown. An OA rat model was established by anterior cruciate ligament transection, and hematoxylin-eosin staining, Safranin-O/Fast Green staining and immunochemistry were applied to estimate OA severity.

**Results:**

IPA did not affect cellular viability at concentrations up to 80 µM. IPA significantly inhibited the IL-1β-induced expression of inflammatory factors (Nitric oxide, PGE2, TNF-α, IL-6, iNOS and COX-2) and matrix-degrading enzymes (MMP-3, MMP-13 and ADAMTS-5), upregulated the expression of anabolic markers (aggrecan and collagen-II) and inactivated the NF-κB pathway. However, AhR knockdown could abolish the above protection capabilities and the suppression of the NF-κB pathway induced by IPA. Furthermore, IPA significantly reduced serum inflammatory cytokines expression, cartilage destruction and synovitis in vivo, demonstrating its protective role in OA progression.

**Conclusion:**

IPA improved IL-1β-induced chondrocyte inflammation and extracellular matrix degradation through the AhR/NF-κB axis, which provides an innovative therapeutic strategy for OA.

**Supplementary Information:**

The online version contains supplementary material available at 10.1186/s10020-023-00614-9.

## Background

Osteoarthritis (OA) is a common age-related joint disease that results in damage to articular cartilage (Hunter and Bierma-Zeinstra 2019). Chronic inflammation plays a central role in OA (Xie and Chen 2019; Robinson et al. 2016). Excessive production of inflammatory cytokines, such as IL-1β, has been implicated in the pathogenesis of OA by mediating chondrocyte inflammation and cartilage degeneration (Wojdasiewicz et al. 2014). It has been revealed that IL-1β can activate the NF-κB signaling pathway and inhibits the expression of matrix synthesis protein including aggrecan and collagen II (Huang et al. 2020, 2021; Varela-Eirin et al. 2018). Therefore, alleviating IL-1β-induced chondrocyte inflammation might provide a potential therapeutic strategy for OA.

In recent years, increasing attention has been paid to the anti-inflammatory properties of gut bacteria metabolites. A latest fecal metabolomics study showed that OA patients have significant perturbations in tryptophan metabolism, particularly the microbial product indole and its metabolites (Rushing et al. 2022). Indole-3-propionic acid (IPA), produced by intestinal flora metabolizing tryptophan, are ligands to activate the aryl hydrocarbon receptor (AhR), which can be absorbed into the blood circulation (Konopelski et al. 2019; Fang et al. 2022). IPA can reduce the expression of pro-inflammatory cytokines (including TNF-α, IL-1β and IL-6) in the liver, colon, astrocytes and muscle cells (Du et al. 2021; Garcez et al. 2020; Rothhammer et al. 2016; Venkatesh et al. 2014; Zhao et al. 2019; Li et al. 2021). Thus, IPA has anti-inflammatory effects in multiple organs, but the role of IPA in inflammatory chondrocytes and OA is not yet clear.

AhR is a ligand-activated transcription factor belonging to the basic helix-loop-helix PER-ARNT-SIM family (Tan et al. 2022). Previous studies suggested that AhR could be triggered by exogenous environmental pollutants and lead to inflammatory responses (Zhang et al. 2022; Quintana and Sherr 2013). However, mounting data indicate that endogenous metabolites can reduce the expression of inflammatory factors by activating AhR (Qiao et al. 2022; Lin et al. 2022; Guerrina et al. 2021; Cui et al. 2022). Furthermore, endogenous ligands for AhR may block the NF-κB signaling pathway and lessen local inflammation in bronchitis, periodontitis and colitis (Yu et al. 2018; Takenaka et al. 2019; Li et al. 2019).

Collectively, we speculate that IPA may reduce chondrocyte senescence and inflammation through the AhR/NF-κB axis. To our knowledge, this is the first study to explore the therapeutic effects of tryptophan metabolites on OA in vivo and in vitro, providing novel insights into the role of intestinal flora and their metabolite IPA in OA treatment.

## Methods

### Animal experiments

A total of fifteen 8-week-old Wistar male rats were provided by the Hubei Provincial Centers for Disease Control and Prevention (Wuhan, Hubei, China). These rats were randomly divided into three groups, namely the sham group, ACLT group and IPA group, with 5 rats per group. The OA rat model in ACLT group and IPA group was established using anterior cruciate ligament transection (ACLT). The rats in the IPA group were then intraperitoneally injected with 20 mg/kg IPA (Aladdin, I103959) twice a week, and rats in the Sham and ACLT group were injected with 50 µL sterile saline as the control. The rats were sacrificed by an injecting of overdose pentobarbital sodium at the end of the 8th week. The animal experiments were performed according to the National Institutes of Health Guide for Care and Use of Laboratory Animals and approved by the Laboratory Animal Welfare & Ethics Committee of the Renmin Hospital of Wuhan University (Approval No: 20220103A).

### Chondrocytes isolation and culture

We isolated cartilage from 8-week-old Wistar male rats to culture primary chondrocytes. The cartilage was cut into 3 mm pieces, then digested for 8 h in DMEM/F12 (Servicebio, G4610) medium containing 0.2% collagenase II (Servicebio, GC305014). Finally, chondrocytes were centrifuged at 1200 rpm for 8 min. The isolated chondrocytes were resuspended in DMEM/F12 medium supplemented with 10% fetal bovine serum (FBS, Gibco, 10270–106) and 1% penicillin-streptomycin (Servicebio, G4003) in a 5% CO_2_ and 37 °C incubator. Plasmids containing short hairpin RNA against AhR (shRNA-AhR) or a scrambled shRNA were obtained from Origene. Chondrocytes were treated with 10 ng/ml IL-1β alone, with IPA, or with shRNA. Transfection was performed by Lipofectamine 3000 (Invitrogen, L3000015) following the manufacturer’s protocol. Subsequent experiments were conducted 48 h after transfection.

### Cell proliferation assay

The cytotoxicity of IPA on chondrocyte viability was quantitated by Cell Counting Kit-8 (CCK-8, Servicebio, G4103). Chondrocytes were seeded in 96-well plates at 5 × 10^3^ cells per well. Then IPA was added at concentrations of 0, 10, 20, 40, 80, 160, 320 and 640 µM. 10 µL of CCK-8 solution was added to each well at 0, 24, 48, 72 and 96 h, and incubated at 37 °C for 2 h. The absorbance at a wavelength of 450 nm was determined using a microplate reader (EnVision, PerkinElmer, USA).

### Real-time quantitative PCR (RT-qPCR)

The total cellular RNA was isolated by the RNA Isolation Kit (Beyotime, R0026) according to the manufacturer’s protocol. The RNA concentration was determined by Nanodrop (Thermo, USA). cDNA synthesis was performed from each 1 µg RNA using SweScript RT II Enzyme Mix (Servicebio, G3330) following the manufacturer’s protocol. RT-qPCR for mRNA was performed on LightCycler 480 (Roche Diagnostics, USA) with 2× Universal SYBR Green Fast qPCR Mix (Abclonal, RK21203). β-actin was used as an internal standard control and relative gene levels were detected by the 2−^△△^Ct method. The primer sequences are shown in Additional file 1: Table S1.

### Western blot

The total proteins from chondrocytes were extracted using radio immunoprecipitation assay buffer, phenylmethanesulfonyl fluoride, phosphatase inhibitors and cotail. Chondrocyte cell lysis was performed for 30 min on ice, and ultrasonication and centrifugation were used to purify proteins. The concentration of proteins was measured by the BCA protein assessment kit. Then, the protein (30 µg) samples were separated on a 10% sodium dodecyl sulfate-polyacrylamide gel. After transferring to PVDF membranes, the membranes were blocked using 5% nonfat milk and then incubated with primary antibodies against IL-6 (1:1000, Abmart, PY6087), iNOS (1:1000, Abclonal, A0312), COX-2 (1:1000, Abclonal, A3560), MMP-3 (1:1000, Abcam, ab52915), MMP-13 (1:1000, Proteintech, 18165-1), ADAMTS-5 (1:1000, Abcam, ab41037), AhR (1:1000, Proteintech, 67785-1), IKKβ (1:1000, Cell Signaling Technology, #2684), p-IKKβ (1:1000, Cell Signaling Technology, #2694), IκBα (1:1000, Abmart, T55026), p-IκBα (1:1000, Abmart, TP56280), p65 (Cell Signaling Technology, 4764), p-p65 (Cell Signaling Technology, 3031) or β-actin (1:3000, Servicebio, GB11001) overnight at 4 ℃. Next, after TBST washed three times, the membranes were incubated with HRP-conjugated secondary antibodies for 2 h at room temperature. After being washed using the TBST buffer, the membrane was incubated with ECL solution and exposed using a Bio-Rad scanner (California, America). Finally, the densitometric analysis was performed using Image-J software.

### Immunofluorescence

Chondrocytes were fixed in 4% paraformaldehyde for 15 min, and washed with PBS three times. The normal goat serum (Servicebio, WGAR1009) was used to block chondrocytes for 30 min at room temperature. The chondrocytes were incubated with primary antibodies against aggrecan (1:500, Servicebio, GB11373) and collagen-II (1:200, Servicebio, GB11021) at 4 °C for 24 h. Then chondrocytes were incubated with fluorescein-conjugated goat anti-rabbit IgG (1:600, Servicebio, GB21301) at 37 °C for 1 h. The nuclei were stained with 4,6-diamidino-2-phenylindole (DAPI). The images were acquired by an inverted fluorescence microscope. Cellular fluorescence intensity was quantified by evaluating positive cells using Image-J software.

### ELISA

Chondrocytes were incubated with 10 ng/ml IL-1β supplemented alone or with IPA or with shRNA-AhR for 24 h. Nitric Oxide (NO), TNF-α, IL-6 and PGE2 expression levels in supernatants were evaluated. Besides, TNF-α, IL-6 and PGE2 expression levels in rat serum were measured. The concentration of NO was assessed using Griess reagent (Beyotime, S0024). The concentration of TNF-α (FineTest, ER1393), IL-6 (elabscience, R0015c) and PGE2 (FineTest, ER1800) levels were measured using ELISA Kits according to the manufacturer’s protocol.

### Bioinformatics data mining

To analyze the binding affinities and modes of interaction between IPA and AhR, AutodockVina 1.2.2, a silico protein-ligand docking software, was employed (http://autodock.scripps.edu/) (Morris et al. 2008). The molecular structure of IPA (PubChem CID: 3744) was retrieved from PubChem Compound (https://pubchem.ncbi.nlm.nih.gov/) (Wang et al. 2017). The 3D structure of AhR (PDB ID: 5NJ8; resolution: 3.3 Å) was downloaded from the PDB (https://www.rcsb.org/). For docking analysis, both protein and molecular files were converted into PDBQT format with all water molecules excluded and polar hydrogen atoms added. The grid box was placed in the center to cover the protein’s domain and accommodate free molecular movement. Molecular docking and visualization were performed by Autodock Vina 1.2.2.

The molecular functional network map of canonical pathways, including physical interactions, co-expression, predicted networks, co-localization, genetic interactions, pathway and shared protein domains of AhR and NF-κB were analyzed using GeneMANIA (http://genemania.org/) (Warde-Farley et al. 2010).

### Histological analysis

Knee joints were isolated from rat and fixed with 4% formaldehyde. Calcium in the knee joints was removed using a decalcifying solution for 28 days. At last, the knee joints were embedded in paraffin and coronally sectioned. The sections were stained with safranin O-fast green or hematoxylin & eosin (H&E) and scored according to the Osteoarthritis Research Society International (OARSI) scoring system to determine the extent of cartilage deterioration (McAlindon 2012). Grade 0 was for intact surface and cartilage; Grade 1 for intact surface only; Grade 2 for surface discontinuity, Grade 3 for vertical fissures; Grade 4 for erosion, Grade 5 for denudation and Grade 6 for deformation.

Histopathological assessment of synovitis was evaluated by enlargement of the synovial lining cell layer and density of the cells. The total synovitis scores were assessed as in the previous study (Lewis et al. 2011). Enlargement of the synovial lining cell layer: 0 Point: Thickness 1–2 cells; 1 Point: Thickness 2–4 cells; 2 Points: Thickness 4–9 cells; 3 points: Thickness ≥ 10 cells. Density of the cells: 0 Point: Synovial stroma shows normal cellularity; 1 Point: Cellularity is slightly increased; 2 Points: Cellularity is moderately increased; 3 Points: Cellularity is greatly increased, pannus formation and rheumatoid-like granulomas might occur.

Immunohistochemistry was conducted with anti-IL-6 (1:100, Abmart, PY6087), anti-MMP-3 (1:50, Abcam, ab52915), anti-MMP-13 (1:200, Proteintech, 18165-1), anti-ADAMTS-5 (1:1000, Abmart, TD13268), anti-aggrecan (1:500, Servicebio, GB11373) and anti-collagen-II (1:100, Servicebio, GB11021).

### Statistics

All results are presented as means with SD. Shapiro-Wilk normality test was used to perform the normality test. For the data with non-normal distribution, log-transformation was performed to transform the data to normal distribution. For the data with normal distribution, Student’s t-test (paired, two groups, equal variances), Welch’s t-test (paired, two groups, unequal variances) and one-way analysis of variance (ANOVA) followed by Bonferroni’s test (multiple groups) were administrated. For rat total synovitis scores and ACLT OARSI scores, a non-parametric test was applied. Statistical analyses were conducted using SPSS version 25 and data was plotted by GraphPad Prism software version 8.0.2). Differences were considered significant when *p* < 0.05.

## Results

### The effect of IPA on chondrocyte viability

**Fig. 1:**
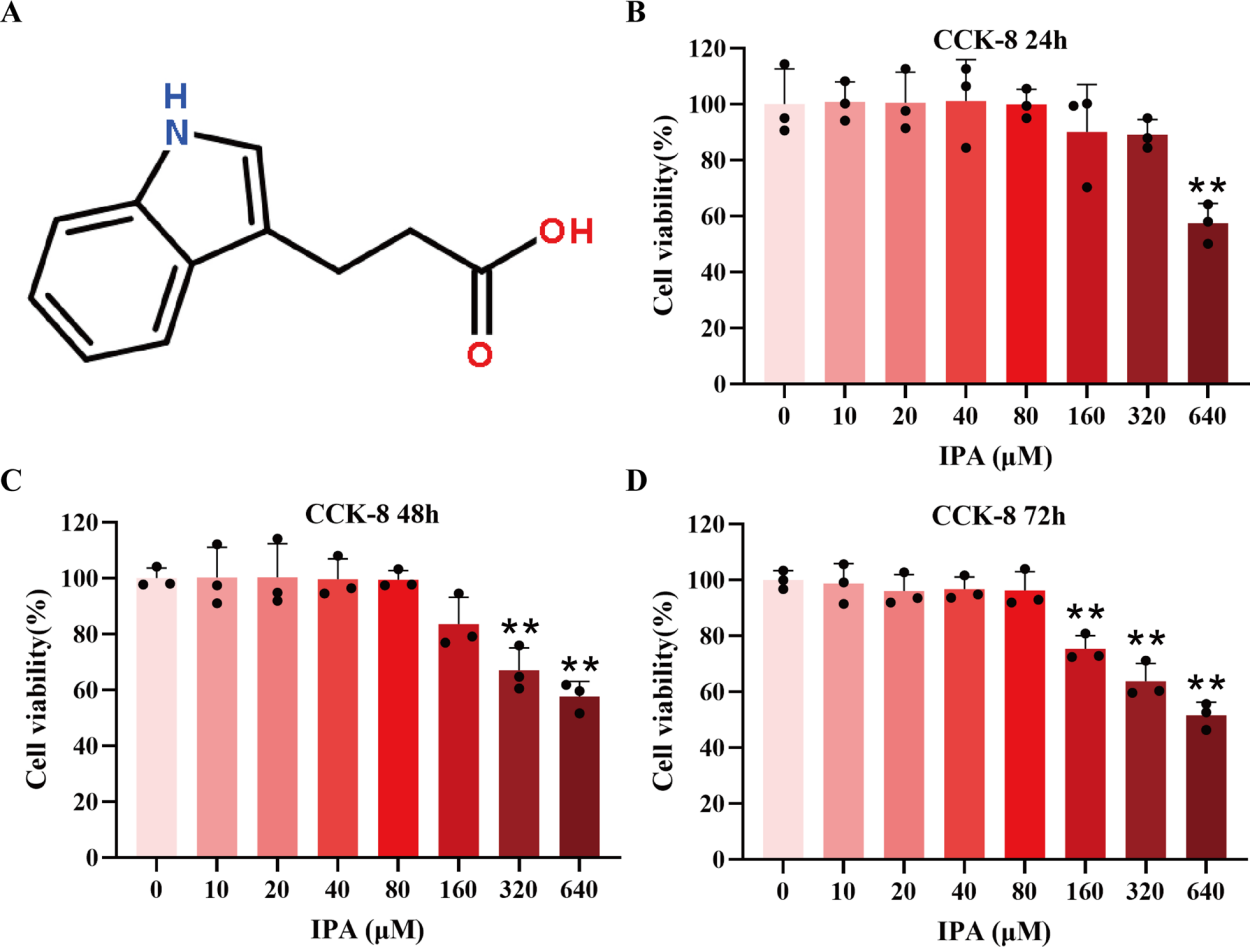
Effects of IPA on chondrocyte viability. **A** Molecular structure of IPA. **B**–**D** Rat chondrocyte viability was evaluated by the CCK-8 assay with indicated concentrations of IPA for 24, 48, and 72 h (n = 3). ^∗^*p* < 0.05, ^∗∗^*p* < 0.01

The molecular structural formula of IPA was shown in Fig. 1A. The rat chondrocytes were pretreated with IPA in concentrations of 0, 10, 20, 40, 80, 160, 320 and 640 µM. Afterward, chondrocyte viability was determined by the CCK-8 assay. As shown in Fig. 1B–D, IPA had no obvious toxicity to rat chondrocytes at 24, 48 and 72 h below the concentrations of 80 µM. Thus, the concentrations of 80 µM IPA were chosen for further in vitro experiments.

### The anti-inflammation ability of IPA

**Fig. 2:**
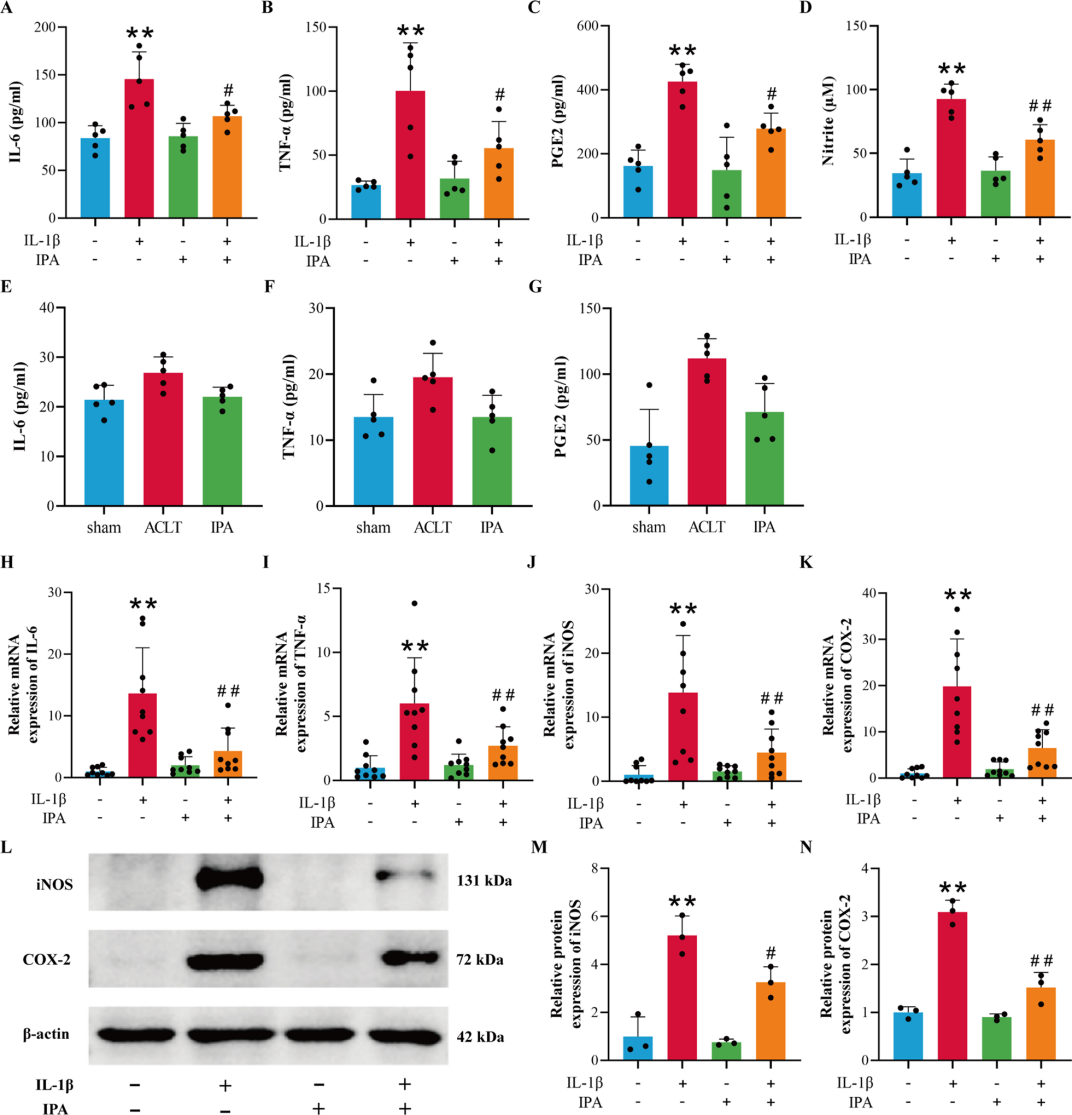
IPA suppressed the production of the inflammatory factors. **A**–**C** Content of IL-6, TNF-α and PGE2 in the cell suspension was detected by ELISA (n = 5). **D** Content of NO was detected by the Griess reaction (n = 5). **E**–**G** Content of IL-6, TNF-α and PGE2 in the rat serum was detected by ELISA (n = 5). **H**–**K** RT-qPCR analysis of IL-6, TNF-α, iNOS and COX-2 in chondrocytes (n = 9). **L**–**N** Western blot and semi-quantitative analysis of iNOS and COX-2 (n = 3). ^*^p < 0.05, ^**^p < 0.01 vs. the control group, ^#^*p* < 0.05, ^##^*p* < 0.01 vs. the IL-1β group

Next, we assessed the protective ability of IPA against IL-1β-induced chondrocyte inflammation. ELISA was carried out to examine the content of IL-6, TNF-α and PGE2 in the cell suspension, whereas NO concentration was determined by the Griess reaction. Compared with the control group, the generation of IL-6, TNF-α, PGE2 and NO were upregulated obviously in IL-1β–induced chondrocytes, while IPA could inhibit the effects of IL-1β (Fig. 2A–D, p < 0.05). To further confirm the anti-inflammation effect of IPA on joint tissues, we built an OA rat model and observed decreased IL-6, TNF-α and PGE2 levels in the serum of rats in the IPA treatment group compared with the ACLT group (Fig. 2E–G, p < 0.05). RT-qPCR results showed that IPA could reverse the induction effects of IL-1β on the mRNA expression of TNF-α, IL-6, COX-2 and iNOS (Fig. 2H–K, p < 0.05). Western blot results revealed that IPA decreased the protein expression of iNOS and COX-2 in IL-1β–activated chondrocytes (Fig. 2L–N, p < 0.05). The above experiments indicated that IPA could reduce the production of inflammatory cytokines.

### The protective effects of IPA on the extracellular matrix in chondrocytes

**Fig. 3:**
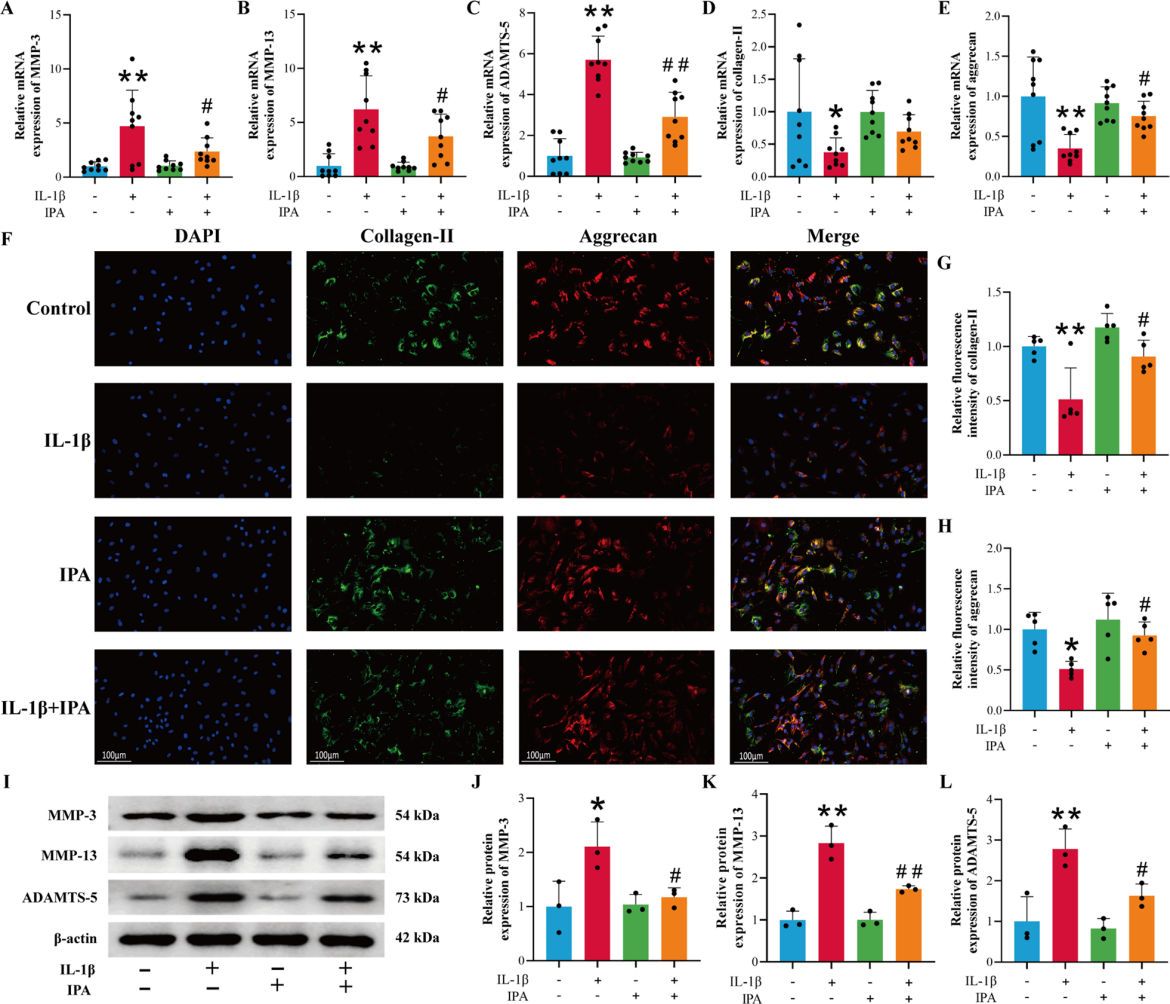
The protective effect of IPA on the IL-1β-induced ECM degradation. **A**–**E** RT-qPCR analysis of MMP-3, MMP-13, ADAMTS-5, collagen-II and aggrecan (n = 9). **F**–**H** Immunofluorescence and semi-quantitative analysis of collagen-II and aggrecan (n = 5, scale bar = 100 μm, ×200). **I**–**L** Western blot and semi-quantitative analysis of MMP-3, MMP-13 and ADAMTS-5 (n = 3). ^*^p < 0.05, ^**^p < 0.01 vs. the control group, ^#^*p* < 0.05, ^##^*p* < 0.01 vs. the IL-1β group

Inflammatory mediators such as NO and PGE2 could promote the secretion of matrix-degrading enzymes such as MMPs and ADAMTS-5. Therefore, the efficacy of IPA on ECM in rat chondrocytes was explored. RT-qPCR results demonstrated that IL-1β increased the mRNA expression of MMP-3, MMP-13 and ADAMTS-5, and decreased the mRNA expression of collagen-II and aggrecan, while IPA could inhibit the effects of IL-1β (Fig. 3A–E, p < 0.05). Immunofluorescence results showed that IPA could reverse the inhibitory effect of IL-1β on collagen-II and aggrecan expression (Fig. 3F–H *p* < 0.05). Western blot results revealed that IPA could reverse the induction effect of IL-1β on catabolic factors-related genes including MMP-3, MMP-13 and ADAMTS-5 (Fig. 3I–L, p < 0.05). The above experiments indicated that IPA could reduce ECM degradation and promote matrix synthesis.

### The protective effects of IPA depend on AhR

**Fig. 4:**
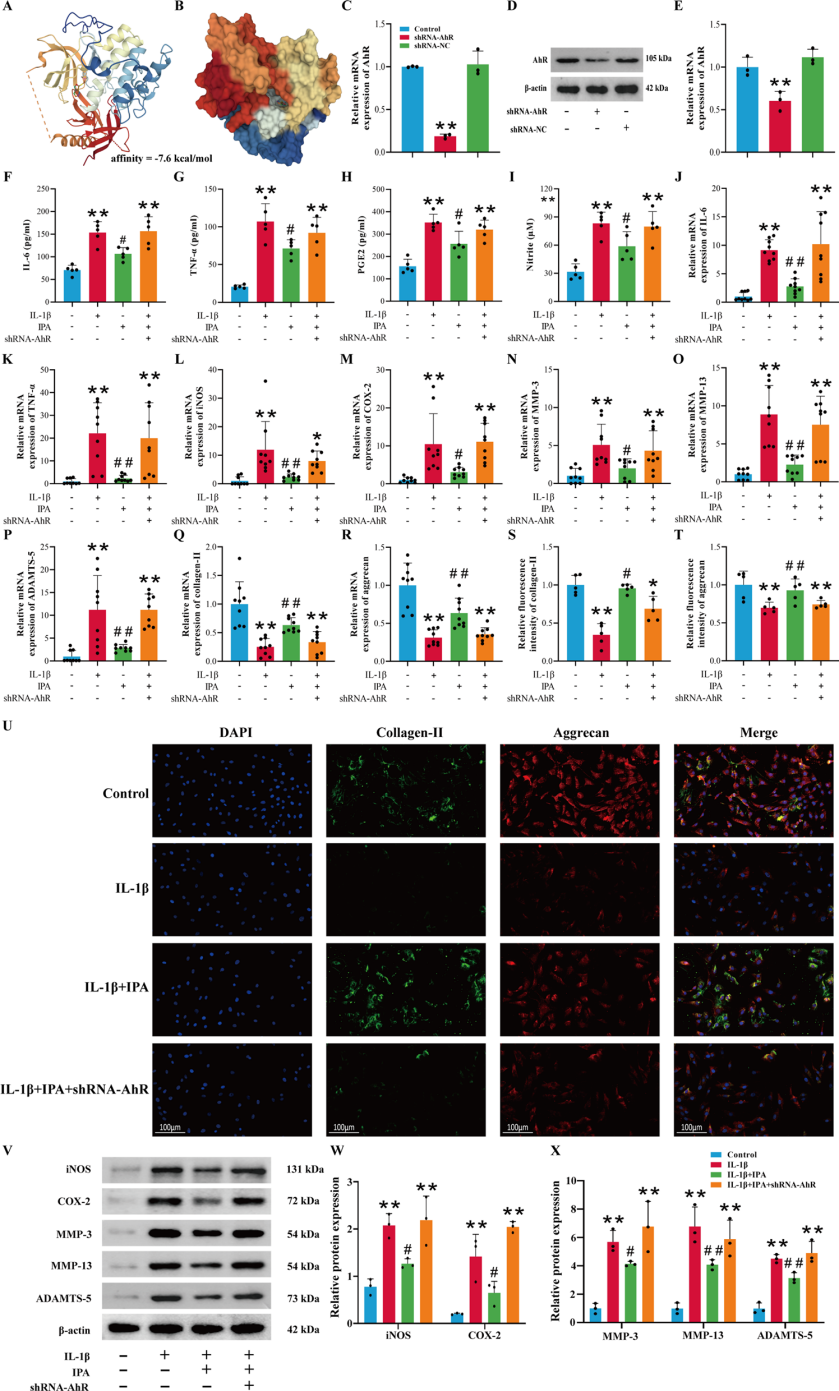
Protective effects of IPA depend on AhR. **A** The binding mode and secondary structure of AhR were demonstrated by a cartoon model. **B** The binding of the AhR pocket was shown with a space-filling model. **C** RT-qPCR analysis of AhR (n = 3); **D**, **E** Western blot and semi-quantitative analysis of AhR (n = 3). **F**–**H** Expression of IL-6, TNF-α and PGE2 was detected by ELISA (n = 5). **I** Expression of NO was detected by Griess reaction (n = 5). **J**–**R** RT-qPCR analysis of IL-6, TNF-α, iNOS, COX-2, MMP-3, MMP-13, ADAMTS-5, collagen-II and aggrecan (n = 9); **S**–**U** Immunofluorescence and semi-quantitative analysis of aggrecan and collagen-II (n = 5, scale bar = 100 μm, ×200); **V**–**X** Western blot and semi-quantitative analysis of COX-2, iNOS, MMP-3, MMP-13 and ADAMTS-5 (n = 3). ^*^p < 0.05, ^**^p < 0.01 vs. the control group, ^#^*p* < 0.05, ^##^*p* < 0.01 vs. the IL-1β group

IPA exerted potent anti-inflammatory capacity through AhR in astrocytes and colon (Rothhammer et al. 2016; Li et al. 2021). To evaluate the affinity of IPA for its target AhR, we performed molecular docking analysis with Autodock Vina. High-affinity (− 7.6 kcal/mol) hydrogen binding events were observed between IPA and AhR (Fig. 4A). Moreover, the space-filling model suggested that IPA was buried within the ligand binding domain (Fig. 4B). Therefore, we suspected that IPA was likely to exert chondroprotective effects via AhR. shRNA-AhR was utilized to verify whether IPA exerted anti-inflammatory capabilities through AhR. The mRNA and protein expression of AhR were both significantly inhibited after chondrocytes were transfected with shRNA-AhR (Fig. 4C–E, p < 0.05). ELISA and Griess reaction results demonstrated that IPA could inhibit the production of IL-6, TNF-α, PGE2 and NO, while these treatment effects were abolished after AhR knockdown (Fig. 4F–I, p < 0.05). RT-qPCR results showed that IPA could reverse the induction effect of IL-1β on IL-6, TNF-α, iNOS, COX-2, MMP-3, MMP-13 and ADAMTS-5, and increase the expression of collagen-II and aggrecan (Fig. 4J–R, p < 0.05). But after AhR knockdown, the above therapeutic effects of IPA disappeared (Fig. 4J–R, p < 0.05). Immunofluorescence results indicated that IPA enhanced the expression of aggrecan and collagen-II (Fig. 4S–U, p < 0.05), and Western blot results also revealed that IPA decreased the expression of COX-2, iNOS, MMP-3, MMP-13 and ADAMTS-5 (Fig. 4V–X, p < 0.05). However, the above effects of IPA were abolished by shRNA-AhR (Fig. 4S–X, p < 0.05). These experiments demonstrated that the protective capabilities of IPA in IL-1β-treated chondrocytes depended on AhR.

### IPA inhibited the NF-κB signaling pathway through AhR

**Fig. 5:**
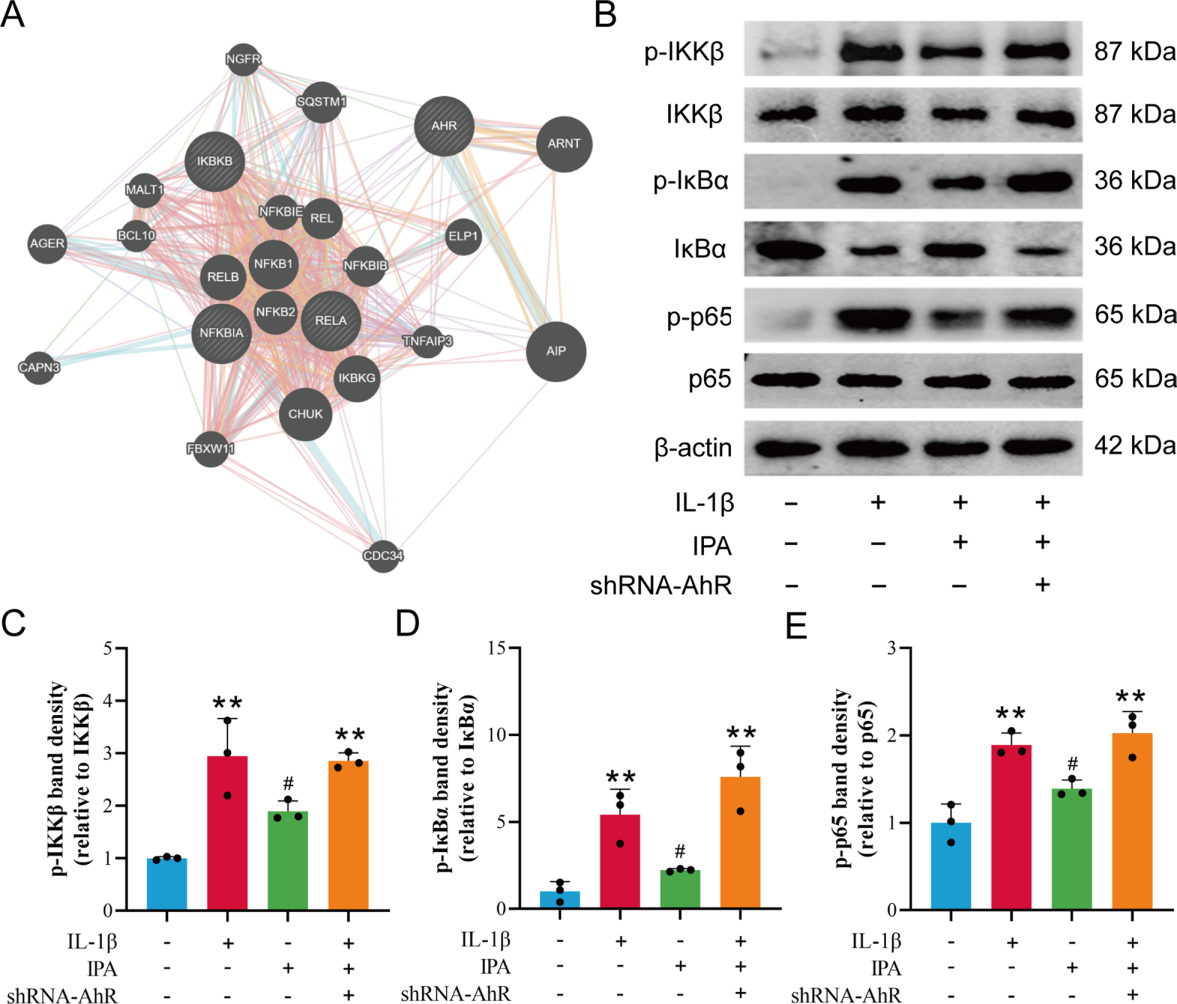
IPA inhibited the NF-κB pathway through AhR. **A** Molecular functional network map of AhR and NF-κB was analyzed using the GeneMANIA tool. **B**–**E** Western blot and semi-quantitative analysis of p-IKKβ, IKKβ, p-IκBα, IκBα, p-p65 and p65 (n = 3). ^**^p < 0.01 vs. the control group, ^#^*p* < 0.05 vs. the IL-1β group

It has been indicated that AhR activated by endogenous ligands could inhibit the NF-κB pathway in bronchitis, periodontitis and colitis (Yu et al. 2018; Takenaka et al. 2019; Li et al. 2019). Therefore, we investigated whether the NF-κB signaling pathway was also involved in the AhR-mediated downstream pathway in chondrocytes. The online GeneMANIA tool was used to observe the possible molecular pathway of AhR and NF-κB pathways. NF-kB subunits [Rela (encoding the p65 subunit), REL, RELB], NFKB inhibitor alpha (IkBα), nuclear factor kappa B subunit 1(NFKB1), NFKB inhibitor epsilon (IKBE), aryl hydrocarbon receptor nuclear translocator (ARNT) and aryl hydrocarbon receptor interacting protein (AIP) were found to be directly associated with AhR (Fig. 5A). Western blot results showed that IPA inhibited IL-1β-induced IKKβ, IκBα and p65 phosphorylation, while shRNA-AhR abolished the inhibitory effect of IPA on the NF-κB pathway (Fig. 5B–E). These findings demonstrated that IPA limited the activation of the NF-κB signaling pathway through AhR.

### IPA attenuated OA progression in the OA rat model

**Fig. 6:**
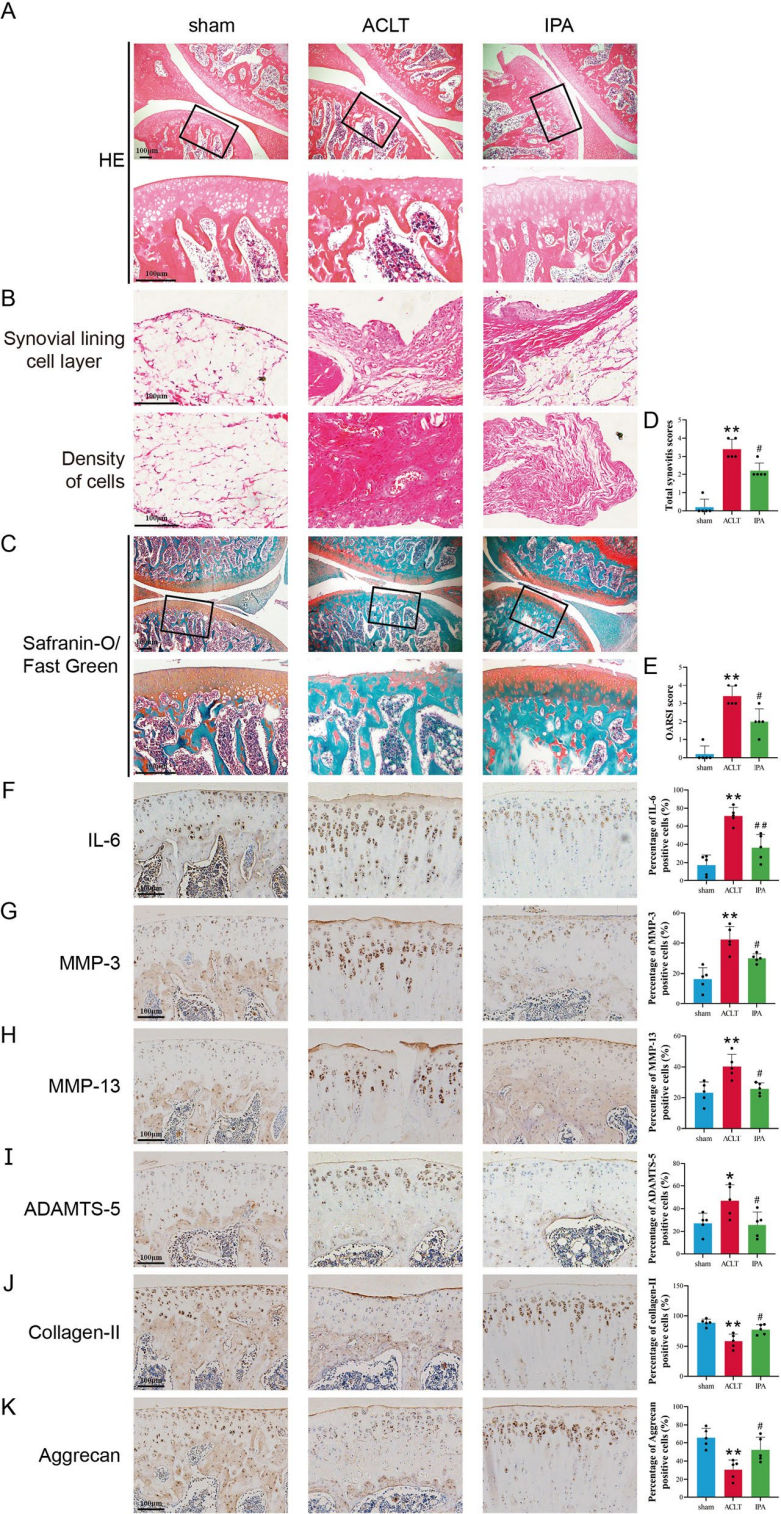
IPA attenuated OA progression in rat model. **A**–**C** Representative images of safranin O-fast green and H&E staining of cartilage and synovial sections from different groups. **D** The total synovitis scores of different groups. **E** The cartilage OARSI scores of different groups. **F**–**K** Immunohistochemistry staining and percentage of positive cells of MMP-3, MMP-13, ADAMTS-5, aggrecan and collagen-II. n = 5, Scale bar = 100 μm. ^**^p < 0.01 vs. the control group, ^##^*p* < 0.01 vs. the ACLT group

To confirm whether IPA played a protective role in OA progression in vivo, the surgical rat OA model was established, and IPA was applied. The results demonstrated that rats in the sham group had smooth cartilage surface while rats in the ACLT group had destructed cartilage surface, eroded cartilage and apparent hypocellularity. Moreover, the synovial inflammation of the ACLT group was more severe than that of the sham group, including enlargement of the synovial lining cell layer and increased density of the resident cells. After IPA treatment, the cartilage degradation and synovial inflammation were alleviated (Fig. 6A–C). Consistent with the observed phenotypes in the cartilage of rats, IPA significantly reduced total synovitis scores and cartilage OARSI score elevated by ACLT (Fig. 6D, E, p < 0.05). Immunohistochemical results showed a significant decrease in IL-6, MMP-3, MMP-13 and ADAMTS-5 expression and a significant increase in aggrecan and collagen-II expression after IPA injection compared with the ACLT group (Fig. 6F–K, p < 0.05). These data demonstrated that IPA efficiently attenuated OA progression in vivo.

## Discussion

The pathogenesis of OA is closely related to sustainable inflammation and the degradation of the chondrocyte ECM. ECM proteins confer biomechanical properties, maintain cell phenotype and mediate tissue repair (Jariwala et al. 2022). Collagen-II and aggrecan are the main components of the chondrocytes ECM (Jariwala et al. 2022). As the characteristic indicators for chondrocytes, collagen-II and aggrecan are necessary to maintain the chondrocyte phenotype. MMP-3, MMP-13 and ADAMTS-5 are the main degrading enzymes of ECM, and the excessive release of these enzymes can induce chondrocytes to lose normal phenotypes (Goldring et al. 2008; Mead and Apte 2018; Milaras et al. 2021). IL-1β can upregulate the expression of iNOS and COX-2 in chondrocytes, and produce NO, PGE2 and TNF-α (Xian et al. 2022). Then, PGE2 and NO could promote the expression of MMP-3, MMP-13 and ADAMTS-5 (Wu et al. 2020). The NF-κB signaling pathway can also be activated by IL-1β, further inducing the excessive production of proinflammatory factors, such as IL-6 and TNF-α (Li et al. 2022). By the introduction of IPA, we found that chondrocyte inflammation and OA progression in the rat model were significantly alleviated. Besides, the elevated expressions of MMP-3, MMP-13 and ADAMTS-5 in the chondrocytes induced by IL-1β were significantly reversed by IPA, indicating a promising inhibitory effect of IPA against the degradation of ECM.

The gut microbiota and its metabolites are deeply involved in the progression of various inflammatory diseases such as osteoarthritis, obesity, ankylosing spondylitis and rheumatoid arthritis (Maeda and Takeda 2017; Costello et al. 2013; Castaner et al. 2018; Chen et al. 2021). Recent studies have demonstrated that Lactobacillus genus was decreased in OA and Lactobacillus genus supplementation improved WOMAC and visual analog scale scores (Chen et al. 2021; Bonato et al. 2022; Lei et al. 2017). Oral administration of Lactobacillus species was effective in alleviating OA symptoms, cartilage destruction, proinflammatory cytokines and macrophage infiltration in a couple of studies (Lei et al. 2017; So et al. 2011; Coulson et al. 2013; Collins et al. 2015; Chen et al. 2020), but the specific therapeutic mechanisms remain to be further investigated. IPA, the major metabolite of Lactobacillus, has anti-inflammatory effects in multiple disease models. Our study found that IPA can reverse IL-1β-induced chondrocytes OA through the AhR/NF-κB axis.

AhR is a ligand-activated transcription factor that induces the expression of multiple downstream genes (Tan et al. 2022). Previous studies suggested that AhR was a receptor for exogenous environmental pollutants such as dioxins, which could mediate inflammatory responses (Zhang et al. 2022; Quintana and Sherr 2013). However, increasing evidence suggested that endogenous metabolites could exert anti-inflammatory effects in immune cells, intestinal epithelial cells, vascular endothelial cells and neuronal cells by activating AhR (Qiao et al. 2022; Lin et al. 2022; Guerrina et al. 2021; Cui et al. 2022). AhR-deficient mice have impaired intestinal barrier function, elevated serum IL-6 levels, and suppressed function of regulatory T cells (Ye et al. 2017; Metidji et al. 2019). These studies revealed that AhR performed a dual role in homeostasis and inflammation. Besides, AhR activation by several endogenous ligands, including 6-Formylindolo(3,2-b)carbazole (FICZ), kynurenine and 1,25-dihydroxyvitamin D3 (VD3), could inhibit the NF-κB signaling pathway and reduce IL-1β expression in periodontitis, glioblastoma and colitis (Yu et al. 2018; Takenaka et al. 2019; Li et al. 2019). AhR activation by FICZ inhibited TNF-α/IFN-γ-induced myosin light chain kinase expression and myosin light chain phosphorylation by suppression of the NF-κB pathway (Yu et al. 2018). Kynurenine produced by glioblastoma cells activated AhR in tumor-associated macrophages and suppressed NF-κB activation (Takenaka et al. 2019). VD3 supplementation enhanced the expression of AhR and reduced alveolar bone loss (Li et al. 2019). Additionally, VD3 decreased NF-κB p65 phosphorylation and inhibited NLRP3, apoptosis-associated speck-like protein, caspase-1, IL-1β and IL-6 expression (Li et al. 2019). Our results identified that endogenous ligand IPA could inhibit IL-1β-induced inflammation and ECM degradation, promote matrix synthesis, and suppress activation of the NF-κB signaling pathway through AhR in chondrocytes.

In the present study, we found that the inflammation, ECM degradation and NF-κB signaling pathway activation induced by IL-1β were significantly suppressed by IPA. When AhR was knocked down in chondrocytes, the protective capabilities of IPA were abolished, indicating that IPA might exert anti-inflammatory effects through AhR. Besides, IPA efficiently attenuated OA progression in the rat model. This report provided novel insights into the therapeutic potentiality of gut microbiota-derived metabolites IPA in OA. In addition, the anti-inflammatory capabilities of AhR activated by endogenous ligands provide a new view for high-throughput screening of small molecule compounds for OA treatment.

This study has a few shortcomings. A major limitation is that both the cell and animal models were derived from rats, and human patient’s primary chondrocyte sample would relate this work to patient-level interventions. Besides, the effects of AhR activation may vary on different cell types, ligands and microenvironments. Therefore, other endogenous ligands of AhR should be explored in future studies.

## Conclusion

In summary, our results demonstrated that IPA reduced IL-1β-induced inflammation and NF-κB signaling pathway through AhR in chondrocytes and efficiently attenuated OA progression in a rat model, which provided a potential treatment strategy for OA.

## Supplementary Information

**Additional file 1: Table S1.** Primer sequences used in this study.

## Data Availability

The datasets used and/or analyzed during the current study available from the corresponding author on reasonable request.

## Abbreviations

OA: Osteoarthritis
IPA: Indole-3-propionic acid
OARSI: Osteoarthritis Research Society International
NO: Nitric oxide
AhR: Aryl hydrocarbon receptor
shRNA-AhR: Short hairpin RNA against AhR
H&E: Hematoxylin & eosin
